# Bioaccessibility and Bioavailability of a Marine-Derived Multimineral, Aquamin-Magnesium

**DOI:** 10.3390/nu10070912

**Published:** 2018-07-17

**Authors:** Valeria D. Felice, Denise M. O’Gorman, Nora M. O’Brien, Niall P. Hyland

**Affiliations:** 1 Department of Pharmacology and Therapeutics and Department of Physiology, University College Cork, T12 K8AF Cork, Ireland; n.hyland@ucc.ie; 2 School of Food and Nutritional Sciences, University College Cork, T12 K8AF Cork, Ireland; nob@ucc.ie; 3 APC Microbiome Ireland, University College Cork, T12 K8AF Cork, Ireland; 4 Marigot Ltd., P43 NN62 Cork, Ireland; denise.ogorman@marigot.ie

**Keywords:** Aquamin, multimineral supplement, magnesium bioavailability

## Abstract

**Introduction:** Magnesium is an essential mineral involved in a range of key biochemical pathways. Several magnesium supplements are present on the market and their degree of bioavailability differs depending on the form of magnesium salt used. Aquamin-Mg is a natural source of magnesium, containing 72 additional trace minerals derived from the clean waters off the Irish coast. However, the in vitro bioaccessibility and bioavailability of Aquamin-Mg in comparison with other supplement sources of magnesium has yet to be tested. **Method:** Aquamin-Mg, magnesium chloride (MgCl_2_) and magnesium oxide (MgO) were subjected to gastrointestinal digestion according to the harmonized INFOGEST in vitro digestion method and in vitro bioavailability tested using the Caco-2 cell model. Magnesium concentration was measured by atomic absorption spectrophotometry (AAS). **Results:** Magnesium recovery from both Aquamin-Mg and MgCl_2_ was greater than for MgO. Magnesium from all three sources was transported across the epithelial monolayer with Aquamin-Mg displaying a comparable profile to the more bioavailable MgCl_2_. **Conclusions:** Our data support that magnesium derived from a marine-derived multimineral product is bioavailable to a significantly greater degree than MgO and displays a similar profile to the more bioavailable MgCl_2_ and may offer additional health benefits given its multimineral profile.

## 1. Introduction 

Magnesium is an essential mineral for the human body and is involved in a wide range of crucial physiological processes [1]. Magnesium can be obtained from the diet, being naturally present in foods such as green leafy vegetables, seeds, beans, whole grains, fish and nuts, amongst others. However, dietary magnesium intake has been shown to be insufficient in the Western population due to industrial food processing that reduces the nutrient contents including magnesium, as well as changes in dietary habits [2]. Deficiency in magnesium dietary intake may lead to hypomagnesemia which has been associated with several disorders including diabetes, osteoporosis and cardiovascular disease [3,4,5]. Early symptoms of hypomagnesemia are non-specific and include loss of appetite, nausea, vomiting, lethargy, fatigue and weakness with more pronounced hypomagnesemia characterised by increased neuromuscular excitability including muscle cramps, tremor, tetany and generalized seizures [6].

The market currently offers various supplement preparations to reach the recommended magnesium daily intake. These supplements differ in the type of magnesium salt used which can be either organic (i.e., magnesium citrate and magnesium aspartate) or inorganic (i.e., MgO and MgCl_2_), their dosage and bioavailability. For example, magnesium from MgCl_2_ has high bioavailability equivalent to organic magnesium supplements such as magnesium lactate and aspartate [7]. Moreover, these three sources of magnesium have significantly greater bioavailability than MgO [7]. Magnesium derived from magnesium hydroxide (Mg(OH)_2_) (Mablet) has been shown to be absorbed into the circulation and, hence, bioavailable in healthy male adults [8]. In a previous study, the bioavailability of magnesium from formulations containing different combinations of magnesium salts displayed similar bioavailability, however the daily dose of magnesium differed [9].

Aquamin-Mg is a natural source of magnesium in the form of Mg(OH)_2_ derived from the clean waters off the Irish coast. In addition to magnesium, Aquamin-Mg also contains 72 additional trace minerals (Marigot Ltd., Cork, Ireland, Table 1) with the same profile of Lithothamnion Aquamin which has previously been shown to be a highly bioavailable source of calcium [10]. 

Here we describe for the first time the in vitro bioaccessibility and bioavailability of Aquamin-Mg in comparison with two commercially available sources of magnesium, MgCl_2_ and MgO.

## 2. Methods 

### 2.1. Harmonized INFOGEST in Vitro Digestion Protocol 

To determine biaccessibility, the amount of magnesium subjected to digestion for each compound was calculated according to the recommended dietary allowance for men (RDA, 420 mg/day). 5.6 mg of magnesium from Aquamin-Mg, MgCl_2_ and MgO were digested according to the harmonized INFOGEST in vitro digestion method published by Minekus and colleagues [11]. Four to five independent digestions were carried out for each compound (Aquamin-Mg, MgCl_2_ and MgO). Aquamin-Mg, MgCl_2_ and MgO were exposed to simulated gastric fluid (composition: 6.9 mM KCl, 0.9 mM KH_2_PO_4_, 25 mM NaHCO_3_, 47.2 mM NaCl, 0.1 mM MgCl_2_(H_2_O)_6_, 0.5 mM (NH_4_)_2_CO_3_). Pepsin and calcium chloride were added to the mixture to achieve a final concentration of 2000 U/mL and 0.075 mM respectively. Hydrochloric acid (HCl, 6 M) was then used to acidify the mixture to pH 3 and water was added to reach a final volume of 20 mL. Samples were then incubated in a stirring water bath at 37 °C and 95 rpm for 2 h. The pH was checked after 1 hour and adjusted if necessary. The simulated intestinal fluid (composition: 6.8 mM KCl, 0.8 mM KH_2_PO_4_, 85 mM NaHCO_3_, 38.4 mM NaCl, 0.33 mM MgCl_2_(H_2_O)_6_) was then added together with pancreatin (the concentration was based on trypsin activity, 100 U/mL) and bile salts for a final concentration of 10 mM. Calcium chloride was also added to achieve a final concentration of 0.3 mM. Sodium hydroxide (NaOH, 1 M) was used to bring the pH to 7 and the necessary amount of water added to reach a final volume of 20 mL. Samples were incubated in a stirring water bath at 37 °C and 95 rpm for 2 h. The pH was checked after one hour and adjusted if necessary. A control sample containing all reagents included in the digestion protocol except the experimental powders was also subjected to the procedure. Upon completion of the incubation period, aliquots (1 mL) of each sample were frozen in liquid nitrogen. Prior to the analysis, one sample from each treatment was filtered using 0.2 μm cell culture sterile filters. The amount of magnesium recovered from these samples was then compared to non-filtered samples.

### 2.2. Caco-2 Cell Bioavailability Assay

Caco-2 cells are human epithelial colorectal adenocarcinoma cells that, upon differentiation, express numerous morphological and biochemical characteristics of small intestinal enterocytes. This in vitro model is widely used to study mineral bioavailability from different sources and their transport mechanisms [12].

For Caco-2 bioavailability experiments 60 mg of magnesium derived from Aquamin-Mg, MgCl_2_ and MgO were subjected to the harmonized INFOGEST in vitro digestion protocol described above (data not shown), and unfiltered samples were used. 60 mg of magnesium was chosen in order to ensure that sufficient concentrations of magnesium could be achieved to perform the Caco-2 experiments. Three independent digestions were carried out for each compound (Aquamin-Mg, MgCl_2_ and MgO) and these were subsequently used in the Caco-2 bioavailability assay. Caco-2 cells (supplied by the European Collection of Authenticated Cell Cultures (ECACC)) were cultured in Dulbecco’s modified eagle’s medium (DMEM) supplemented with 1% non-essential amino acids and 10% foetal bovine serum (FBS) and were stored in a humidified incubator at 37 °C and 5% CO_2_. For all experiments, cells were seeded at a density of 1 × 10^5^ cells/mL on 6-well Transwell plates with inserts of 24 mm diameter and differentiated for 21 days. Media was changed every other day.

### 2.3. Bioavailability of Magnesium from Aquamin-Mg, MgCl_2_ and MgO Using Caco-2 Cells

On the day of the experiment, media was removed from all wells and 1 mL of fresh media was added to the luminal side and 2 mL to the basolateral side of each well. Transepithelial electrical resistance (TEER) was measured to confirm the integrity of the epithelial monolayer. Cells were then incubated with either Aquamin-Mg, MgCl_2_- or MgO-derived magnesium in the luminal side (concentrations of 25, 50, 100 and 150 μg/mL) for 2 h at 37 °C. Samples from each independent digestion were used in a corresponding independent bioavailability study which was conducted in triplicate. Two controls were included in the assay, a blank sample, only containing media, and a digest sample, containing all reagents included in the digestion protocol except the experimental powders. At the end of the incubation time, TEER values were recorded again to ensure that the treatments did not have any effect on the integrity of the monolayer. Luminal and basolateral samples were collected and stored at 4 °C. Magnesium concentration of luminal and basolateral samples was measured by atomic absorption spectrophotometry (AAS). Three independent experiments were carried out. Each treatment was randomly assigned and was performed in duplicate.

### 2.4. Atomic Absorption Spectrophotometry (AAS)

The magnesium content of the digested samples, as well as luminal and basolateral samples was determined by AAS. Samples were diluted in Milli-Q water prior to analysis. Lanthanum chloride (0.1%) was also added to eliminate any phosphate interferences. A commercially available magnesium standard (Spectrosol from BDH Chemicals Ltd., Dublin, Ireland) was used. Standard solutions were prepared using Milli-Q water containing lanthanum chloride (0.1%) and ranged from 0 to 1 mg/L.

### 2.5. Statistical Analyses

Data are expressed as mean ± standard error of the mean (SEM). Statistical analysis was carried out using the Kruskal–Wallis test, followed by Dunn’s multiple comparison test for the digestion study. For the bioavailability study, we calculated the residuals of the data to determine whether there were outliers and statistical analysis was performed using one-way analysis of variance (ANOVA) followed by Bonferroni post-hoc test. Values of *p* < 0.05 were considered statistically significant.

## 3. Results

### 3.1. Magnesium Recovery from Aquamin-Mg, MgCl_2_ and MgO Following In Vitro Digestion

**Figure 1:**
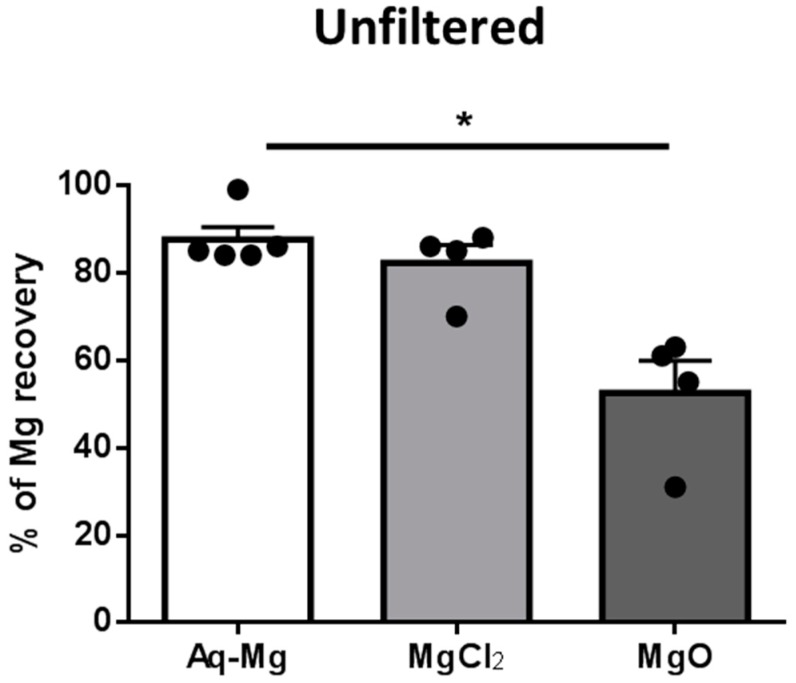
Magnesium recovery from Aquamin-Mg, MgCl_2_ and MgO following in vitro digestion. The percentage of magnesium recovery from Aquamin-Mg was significantly higher than MgO (* *p* < 0.5, *n* = 4–5) in unfiltered samples.

Aquamin-Mg-derived magnesium, showed an in vitro bioaccessibility more similar to highly soluble alternative such as MgCl_2_ than to MgO (Aquamin-Mg, 122.6 ± 4.1 μg/mL, *n* = 5; MgCl_2_, 115.4 ± 6.0 μg/mL, *n* = 4; MgO, 73.39 ± 10.20 μg/mL, *n* = 4; in unfiltered samples), which is characterised by low solubility (Figure 1, *p* < 0.05 Aquamin-Mg vs. MgO). To determine whether magnesium was lost during filtration for the Caco-2 cell culture experiments, magnesium concentration in filtered samples was also determined and these were lower for all the treatments relative to unfiltered samples (Aquamin-Mg, 99.3 ± 6.2 μg/mL, *n* = 5; MgCl_2_, 92.2 ± 5.6 μg/mL, *n* = 4; MgO, 64.5 ± 9.3 μg/mL in filtered samples).

### 3.2. Transepithelial Electrical Resistance (TEER)

**Figure 2:**
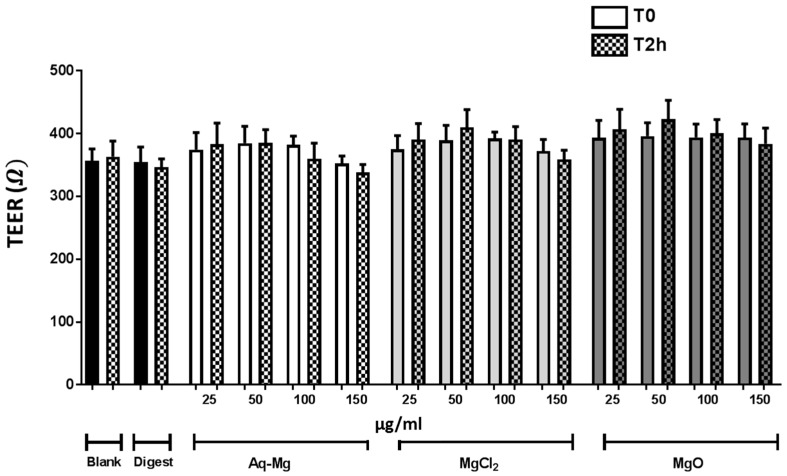
Transepithelial electrical resistance in Caco-2 cells following treatment with Aquamin-Mg, MgCl_2_ and MgO. At time zero (T0) cells were differentiated in an integral monolayer. Transepithelial electrical resistance (TEER) values confirmed that the treatments did not compromise the integrity of the monolayer at any of the concentrations tested following 2 h incubation (T2h) (*n* = 3).

As an indicator of cell viability we measured TEER at the start of each experiment and after treatment. The results from the transepithelial resistance measurements confirmed that after 21 days of culture, Caco-2 cells formed an integral monolayer. Moreover, none of the treatments, at any concentration tested, affected epithelial integrity following 2 h incubation at 37 °C (Figure 2). 

### 3.3. Bioavailability of Magnesium from Aquamin-Mg, MgCl_2_ and MgO Using the Caco-2 Cell Model

**Figure 3:**
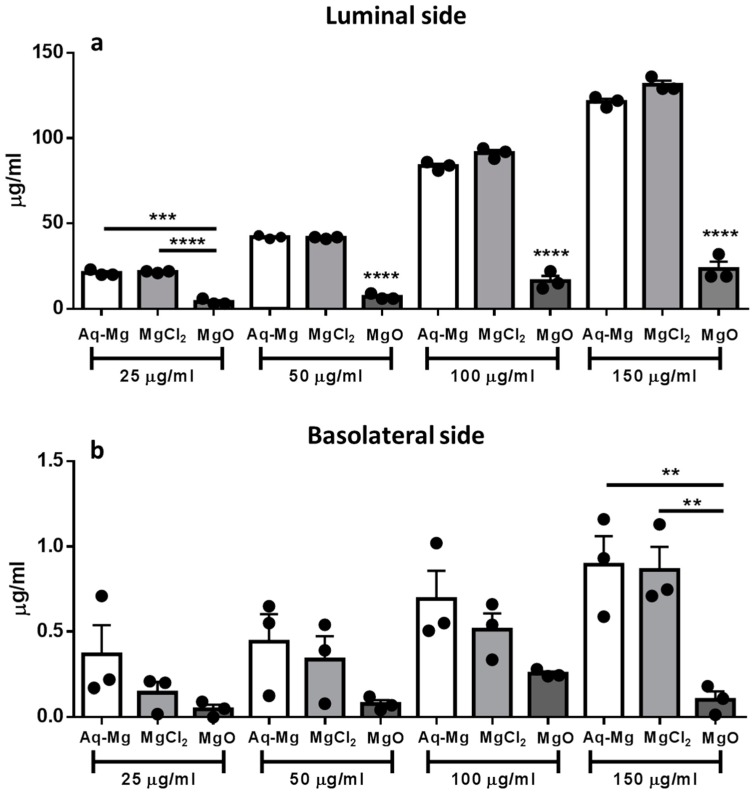
Bioavailability of magnesium from Aquamin-Mg, MgCl_2_ and MgO using Caco-2 cells. (**a**) Magnesium derived from Aquamin-Mg, MgCl_2_ and MgO applied into the luminal side. Following in vitro digestion, the amount of bioaccessible magnesium was significantly higher for Aquamin-Mg and MgCl_2_ compared to MgO (*** *p* < 0.001 for Aquamin-Mg vs. MgO at 25 μg/mL, **** *p* < 0.0001 for MgCl_2_ vs. MgO at 25 μg/mL and **** *p* < 0.0001 for Aquamin-Mg and MgCl_2_ vs. MgO at 50, 100 and 150 μg/mL, *n* = 3); (**b**) Magnesium derived from Aquamin-Mg, MgCl_2_ and MgO measured in the basolateral side after 2 h incubation at 37 °C. At the highest concentration tested, magnesium from Aquamin-Mg and MgCl_2_ was significantly more bioavailable than MgO (** *p* < 0.01 for Aquamin-Mg and MgCl_2_ vs. MgO at 150 μg/mL, *n* = 3).

Digestates were added to the luminal compartment. The reduced amount of magnesium from MgO is reflective of the reduced bioaccessibility (Figure 3a). These results are in accordance with the in vitro digestion data (60 mg; data not shown). When the concentration of magnesium was measured in the basolateral chamber, magnesium derived from Aquamin-Mg and MgCl_2_ showed the same degree of bioavailability at all the concentrations tested, while only a small amount of magnesium from MgO was transported across the epithelium and thus bioavailable. Both Aquamin-Mg and MgCl_2_ were significantly more bioavailable than MgO at the highest concentration tested (150 μg/mL) (Figure 3b, Table 2).

## 4. Discussion

The aim of these studies was to examine the bioaccessibility and bioavailability of magnesium from Aquamin-Mg compared to MgCl_2_ and MgO using the Caco-2 cell model. In this model both active saturated and passive non-saturated pathways have been previously identified for magnesium transport [13]. The study from Thongon and Krishnamrain has indeed shown that in Caco-2 monolayers, magnesium transported from the apical to the basolateral side (representing magnesium absorption) against magnesium in the apical solution (representing magnesium concentration) was curvilinear as previously shown in humans [13,14]. Furthermore, the same study has shown that treatment with omeprazole selectively inhibited the non-saturable passive component, without affecting the saturable active component of magnesium transport which was abolished using the Transient Receptor Potential Cation Channel Subfamily M Member 6 (TRPM6) inhibitor Ruthenium Red (RR) [13]. This evidence shows that magnesium can be transported through both a paracellular and a transcellular pathway and that the Caco-2 monolayer is a suitable in vitro model of intestinal magnesium absorption. In the context of our findings, however, we cannot comment on which pathway was responsible for the apical to basolateral transport of magnesium and further research is warranted in order to elucidate these mechanisms. Our results show for the first-time, however, direct evidence that Aquamin-Mg-derived magnesium is highly bioaccessible following in vitro digestion and magnesium is transported across the intestinal epithelium in this well-established in vitro model. Moreover, the degree of bioaccessbility and bioavailability of Aquamin-Mg was comparable to MgCl_2_ while being superior to MgO.

MgCl_2_ and MgO represent a high bioavailable and low bioavailable source of magnesium respectively, and our in vitro data are in keeping with in vivo data demonstrating that the mean urinary excretion of magnesium in healthy volunteers was significantly higher for MgCl_2_ than MgO [7]. Interestingly, in this study, MgCl_2_ bioavailability was comparable to that of organic magnesium forms such as magnesium aspartate and magnesium lactate [7]. 

Magnesium in Aquamin-Mg is in the form of Mg(OH)_2_. However, as well as magnesium, Aquamin-Mg also provides 72 additional trace minerals all derived from sea water (Marigot Ltd., Cork, Ireland, Table 1). In support of Mg(OH)_2_ as a magnesium supplement, the pharmacokinetic profile of a single oral dose of Mg(OH)_2_ in healthy male adults showed that the bioavailability of magnesium from Mg(OH)_2_ was 15% [8]. Moreover, none of the participants recruited reported any side effect following supplementation suggesting that Mg(OH)_2_ may be a clinically relevant option for oral magnesium supplementation [8]. In a second human study the degree of bioavailability of Mg(OH)_2_ was compared to other sources of magnesium, including MgCl_2_ measured as urinary elimination of magnesium [9]. In this study it was found that Mg(OH)_2_ was required at a higher dose to reach the same level of bioavailability [9]. 

The solubility of magnesium in the gastrointestinal tract plays a key role in magnesium absorption. Our bioaccessiblity results demonstrate that magnesium from Aquamin-Mg is soluble as MgCl_2_ and hence potentially available for absorption. Our bioavailability data support that Mg(OH)_2_, derived from Aquamin-Mg, displays a similar profile and transport characteristics as magnesium derived from MgCl_2_ at the same concentrations, suggesting that Aquamin-Mg represents a source of magnesium coupled with potential health benefits of a multimineral supplement. Currently, however, as Aquamin-Mg is not formulated as an oral supplement (tablets and capsules), further comparisons with other formulated magnesium supplements were not possible.

Moreover, Aquamin-Mg is composed of multiple minerals and whether these affect its bioaccessibility or bioavailability is difficult to determine.

## 5. Conclusions

In conclusion, our data suggests that Aquamin-Mg-derived magnesium is bioaccessible and bioavailable to a significantly greater degree than magnesium oxide while displaying a comparable profile to magnesium chloride. Nonetheless, our in vitro results are qualitatively consistent with the clinical study from Firoz and Graber showing that magnesium from MgCl_2_ has significantly greater bioavailability than MgO. Further research is warranted to investigate the bioaccessibility and bioavailability of Aquamin-Mg in clinical studies. 

## Figures and Tables

**Table 1:** Mineral composition of Aquamin-Mg. (ppm, parts per million).

Mineral	ppm	Mineral	ppm
Aluminum	461	Molybdenum	1.78
Antimony	<0.5	Neodymium	2.810
Arsenic	1.811	Nickel	<0.5
Barium	1.83	Niobium	<0.5
Beryllium	<0.5	Osmium	0.002
Bismuth	<0.5	Palladium	0.301
Boron	186	Phosphorus	117
Cadmium	0.394	Platinum	0.002
Calcium	23,000	Potassium	88.21
Carbon	10,100	Praseodymium	0.697
Cerium	3.411	Rhenium	0.001
Cesium	0.008	Rhodium	0.010
Chloride	613.4	Rubidium	0.063
Chromium	5.83	Ruthenium	0.135
Cobalt	<0.5	Samarium	0.576
Copper	5.09	Scandium	1.050
Dysprosium	0.829	Selenium	<0.5
Erbium	0.613	Silicon	657
Europium	0.197	Silver	<0.5
Fluoride	1.1	Sodium	1467
Gadolinium	0.770	Strontium	84.7
Gallium	0.163	Sulfur	3335
Germanium	0.020	Tantalum	0.016
Gold	<0.5	Tellurium	<0.5
Hafnium	0.046	Terbium	0.140
Holmium	0.194	Thallium	<0.5
Indium	<0.001	Thorium	0.860
Iodine	9.1	Thulium	0.081
Iridium	0.002	Tin	0.179
Iron	1213	Titanium	18.5
Lanthanum	1.01	Tungsten	2.08
Lead	0.604	Vanadium	16.0
Lithium	<0.5	Ytterbium	0.498
Lutetium	0.116	Yttrium	7.38
Magnesium	404,400	Zinc	2.37
Manganese	486	Zirconium	<0.5
Mercury	0.009		

**Table 2:** Magnesium derived from Aquamin-Mg, MgCl_2_ and MgO measured in the basolateral side after 2 h incubation at 37 °C expressed as μg/mL (*n* = 3).

Magnesium Source	Sample Concentration (μg/mL)	Basolateral Side μg/mL Mean ± Standard Error of the Mean (SEM)
Aquamin-Mg	25	0.37 ± 0.17
	50	0.44 ± 0.16
	100	0.69 ± 0.16
	150	0.89 ± 0.17
MgCl_2_	25	0.14 ± 0.06
	50	0.33 ± 0.13
	100	0.51 ± 0.09
	150	0.86 ± 0.13
MgO	25	0.05 ± 0.03
	50	0.08 ± 0.02
	100	0.25 ± 0.01
	150	0.10 ± 0.05
